# A new scheme to discover functional associations and regulatory networks of E3 ubiquitin ligases

**DOI:** 10.1186/s12918-015-0244-1

**Published:** 2016-01-11

**Authors:** Kai-Yao Huang, Julia Tzu-Ya Weng, Tzong-Yi Lee, Shun-Long Weng

**Affiliations:** Department of Computer Science and Engineering, Yuan Ze University, Taoyuan, 320 Taiwan; Innovation Center for Big Data and Digital Convergence, Yuan Ze University, Taoyuan, 320 Taiwan; Department of Obstetrics and Gynecology, Hsinchu Mackay Memorial Hospital, Hsin-Chu, 300 Taiwan; Mackay Junior College of Medicine, Nursing and Management, Taipei, 112 Taiwan; Department of Medicine, Mackay Medical College, New Taipei City, 252 Taiwan

**Keywords:** Ubiquitination, Ubiquitin, E3 ubiquitin ligase, Protein-protein interaction, Ubiquitination network

## Abstract

**Background:**

Protein ubiquitination catalyzed by E3 ubiquitin ligases play important modulatory roles in various biological processes. With the emergence of high-throughput mass spectrometry technology, the proteomics research community embraced the development of numerous experimental methods for the determination of ubiquitination sites. The result is an accumulation of ubiquitinome data, coupled with a lack of available resources for investigating the regulatory networks among E3 ligases and ubiquitinated proteins. In this study, by integrating existing ubiquitinome data, experimentally validated E3 ligases and established protein-protein interactions, we have devised a strategy to construct a comprehensive map of protein ubiquitination networks.

**Results:**

In total, 41,392 experimentally verified ubiquitination sites from 12,786 ubiquitinated proteins of humans have been obtained for this study. Additional 494 E3 ligases along with 1220 functional annotations and 28588 protein domains were manually curated. To characterize the regulatory networks among E3 ligases and ubiquitinated proteins, a well-established network viewer was utilized for the exploration of ubiquitination networks from 40892 protein-protein interactions. The effectiveness of the proposed approach was demonstrated in a case study examining E3 ligases involved in the ubiquitination of tumor suppressor p53. In addition to Mdm2, a known regulator of p53, the investigation also revealed other potential E3 ligases that may participate in the ubiquitination of p53.

**Conclusion:**

Aside from the ability to facilitate comprehensive investigations of protein ubiquitination networks, by integrating information regarding protein-protein interactions and substrate specificities, the proposed method could discover potential E3 ligases for ubiquitinated proteins. Our strategy presents an efficient means for the preliminary screen of ubiquitination networks and overcomes the challenge as a result of limited knowledge about E3 ligase-regulated ubiquitination.

**Electronic supplementary material:**

The online version of this article (doi:10.1186/s12918-015-0244-1) contains supplementary material, which is available to authorized users.

## Introduction

Protein ubiquitination involves a series of enzymatic reactions such as E1 activation, E2 conjugation, and E3 ligation, resulting in the conjugation of single or multiple ubiquitin proteins at a target lysine residue [1]. Numerous substrate proteins with ubiquitination sites have been characterized to date, owing to the emergence of high-throughput mass spectrometry-based proteomics approaches [2–4]. Identified to play key roles in transcriptional regulation, signal transduction, development, apoptosis, endocytosis, cell proliferation and cancers, ubiquitination of the lysine residue has been regarded as an essential mediator of various biological processes [5–7]. Among the enzymes that catalyze protein ubiquitination, E3 ligases are particularly important for the recognition of substrate sites to facilitate ubiquitin-mediated protein degradation [8]. The relationships between E3 ligase and substrates are complex. Multiple substrates could be targeted by a single E3 ligase; alternatively, multiple E3 ligases could catalyze the ubiquitination of a single substrate [9]. These substrate-enzyme correlations could be used to construct E3-specific regulatory networks and map to the associated cellular pathways, making possible the characterization of complex cellular processes and functional analysis of E3-sbustrate relationships [9]. This approach has allowed the discovery of the role that anaphase-promoting complex (APC)/cyclosome plays in modulating key targets of the cell cycle, such as cyclins and their related E3 ligases [10–12].

To date, a significant amount of research efforts have been invested towards the characterization of E3 structures and examination of the mechanisms underlying E3-mediated regulatory networks, as well as E3-related diseases [13–21]. Based on their catalytic mechanisms in the ubiquitination process, E3 ligases can be classified into three major types: the HECT (homologous to E6-AP C-terminus), the RING (really interesting new gene), and U-box domain types [22]. The HECT-type is responsible for catalyzing the attachment of ubiquitin to substrate proteins. In contrast, the RING-type and U-box-type, similar in both structure and function, facilitate the interaction between an E2 enzyme and the target proteins. Regardless of the types, the significance of E3-mediated ubiquitination is obvious from their association with diseases [23]. Several studies have suggested that the inhibition of E3 ligases may cause growth suppression or cell death, as evidenced by the over-expression of Mdm2/Hdm2, IAPs, and SCF in various human cancers [24]. Therefore, regulation of E3 ligase activities and functions may be a promising approach for cancer treatments.

Many databases and tools have been developed to aid in the study of E3 ligases. For example, E3Miner [25] offers a text mining approach to identify ubiquitin-protein ligases, whereas E3Net [9] allows users to search through a collection of 1671 E3-substrate relationships among 493 E3s and 1277 substrates in 42 organisms. In contrast, by analyzing protein sequence similarities, domains, and distributions across different species, Sakiyama et al*.* [26] constructed a useful database for the exploration of proteins involved in the ubiquitin signaling cascade. Unfortunately, the present accumulation of large-scale ubiquitinome data demands for the development of tools that investigate the regulatory networks of E3 ligases and their substrates. Here, we present a new strategy that utilizes an interactive network viewer to assist with the discovery of novel protein ubiquitination networks. Furthermore, to effectively investigate the relationships between E3 ligases and their substrates, metabolic pathways and protein-protein interactions (PPIs) were integrated to construct comprehensive protein ubiquitination networks. The ability of the proposed method to identify E3 ligase-mediated ubiquitination networks and their biological significance was demonstrated by case studies. The results indicated that, despite the current limited knowledge about regulatory relationship between E3 ligases and ubiquitinated proteins, our approach could uncover potential E3 ligase-substrate relationships based on based on protein-protein interaction information and substrate site specificities.

## Materials and method

**Fig. 1:**
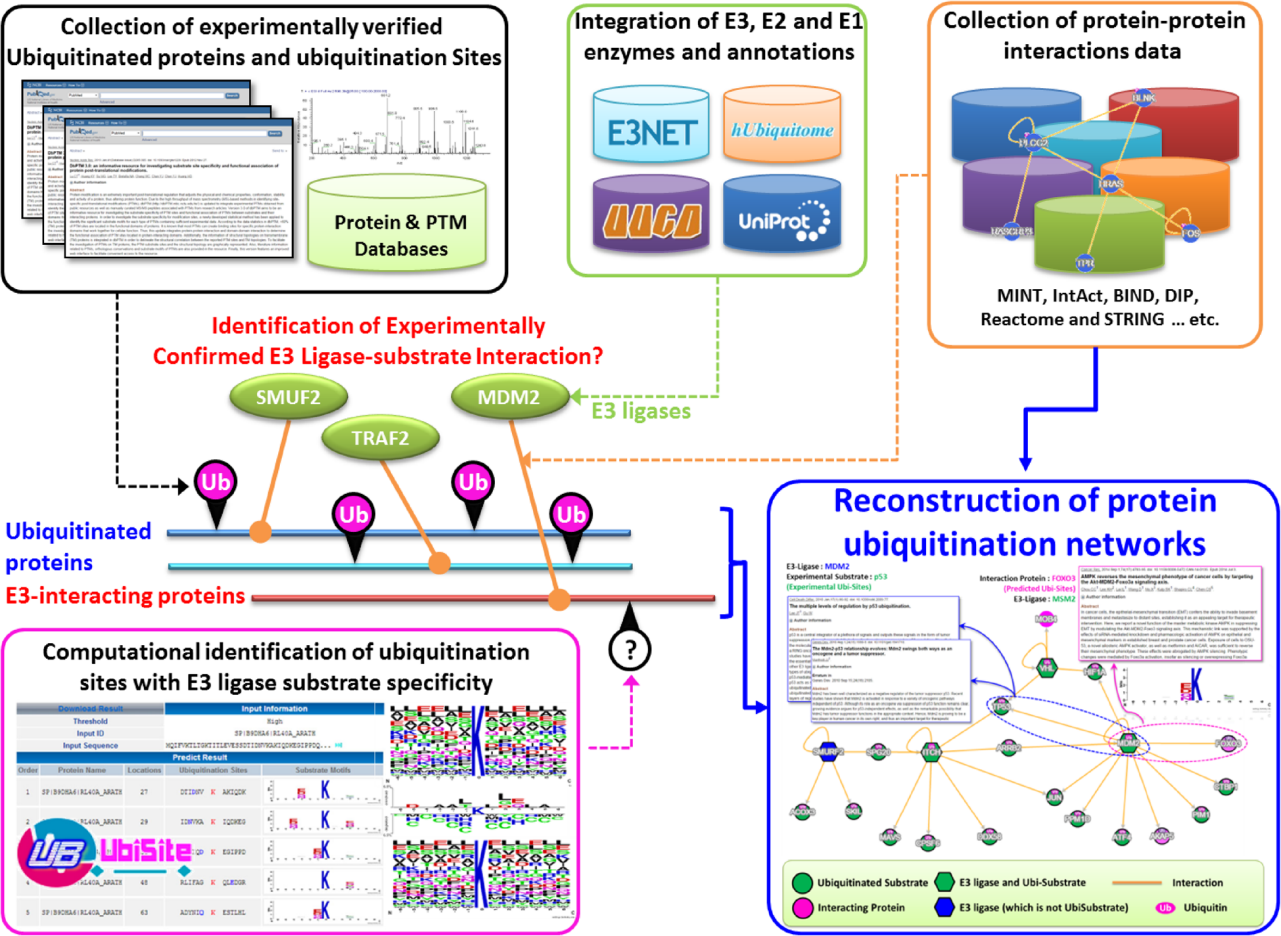
System flow of protein ubiquitination network construction

Construction of the protein ubiquitination networks involved collection of E3 ligase and ubiquitinated protein data, integration of ubiquitinated proteins’ functional data, computational identification of ubiquitination sites based on substrate motifs, as well as network construction using protein-protein interactions and metabolic pathways (Fig. 1). A network viewer was employed to provide a visualization of the ubiquitination regulatory network, with implemented functional information, for a group of proteins of interest. The detailed workflow is described as follows.

### Data collection of E3 ubiquitin ligases and ubiquitinated proteins

Experimentally validated E1 activating, E2 conjugating, and E3 ligating enzyme data were obtained from various sources. From UUCD-Version 1.0 [27], seven distinct E1 activating enzymes were collected. From E3Net, UUCD [27], hUbiquitome [28], and UniProtKB [29], 494 non-redundant E3 ubiquitin ligases and their biological functions were extracted. In addition, a total of 46 non-redundant E2 conjugating enzymes were collected from UUCD [27], hUbiquitome [28] and UniProtKB [29]. Experimentally verified ubiquitination sites from dbPTM [30–32] were also included. Next, search keywords, such as “ubiquitinated”, “ubiquitination”, “ubiquitylated”, or “ubiquitylation”, were entered on the PubMed database to extract ubiquitinated protein data from research articles. Specifically, full texts of the matched research articles were manually reviewed to ensure that the exact ubiquitinated peptide and modified lysine residue information were extracted. Finally, redundant data were removed, generating a total of 41,392 ubiquitinated lysines from 12,786 ubiquitinated human proteins.

### Characterization of protein ubiquitination sites

To characterize the amino acid composition of protein ubiquitination sites, WebLogo [33, 34] was utilized to generate the relative frequency of the corresponding amino acid at each position around the ubiquitination sites as represented by the graphical sequence logo. As well, to further discriminate the amino acid composition of ubiquitinated sites from their non-ubiquitinated counterparts, TwoSampleLogo [35] was adopted to display statistically significant differences in position-specific symbol compositions. The inherent complexity of large-scale ubiquitinome data may make it difficult to uncover conserved motifs. To overcome this problem, MDDLogo [36] was applied to identify potential motifs for the curated protein ubiquitination sites. MDDLogo is a program that uses the maximal dependence decomposition (MDD) approach to discover conserved motifs from groups of aligned signal sequences through a recursive process that divides the data sets into tree-like subgroups. The effectiveness of MDDLogo has been demonstrated in the identification of substrate motifs for phosphorylation [37–40], *S*-nitrosylation [41], O-GlcNAcylation sites [42], *S*-glutathionylation [43], as well as ubiquitin conjugation sites [2].

### Data integration for functional investigation of ubiquitinated proteins

To investigate the biological significance of ubiquitinated proteins, various biological databases, such as Gene Ontology (GO) [44], InterPro [45], as well as KEGG Diseases and Pathways [46], were incorporated. To provide comprehensive functional annotations of proteins associated with ubiquitination, the ubiquitinated proteins were classified according to their molecular functions, biological processes, and cellular components. Since ubiquitination is known to regulate the cellular localization, interactions, and degradation of proteins [47–49], the biological roles of ubiquitination sites within a specific protein domain could be inferred from the functional annotation of the domain. For this purpose, essential protein family, domain, and functional site information was obtained from InterPro [45], a database which integrates data from various sources such as the PROSITE [50], PRINTS [51], Pfam [52], and ProDom [53].

### Network construction using protein-protein interactions and metabolic pathways

Substantial evidence supports the role that protein ubiquitination plays in the regulation of cellular processes. Thus, by integrating experimentally validated mammalian E3 ubiquitin ligases and their functional information, we hoped to provide a foundation for navigating ubiquitination regulatory networks in mammals. To facilitate the exploration of regulatory relationships between E3 ligases and their ubiquitinated substrates, associated metabolic pathways and protein-protein interactions (PPIs) were included for the comprehensive construction of protein ubiquitination networks. The human metabolic pathways were extracted from KEGG [54]. Experimentally verified PPIs were obtained from over ten PPI databases (Additional file 1: Table S1). Potential PPIs predicted based on co-regulation, co-occurrence in the literature, co-expression, and genomic context were curated from the STRING database [55]; each interaction included a confidence score calculated by the STRING built-in function.

Next, a graph theory [56, 57] approach has been adopted to illustrate the relationships between E3 ligases and substrates. Specifically, we use a directed and cyclic graph *G* = ( *V* , *E* ) to symbolize a protein ubiquitination network, where *x* , *y* ∈ *V* and ( *x* , *y* ) ∈ *E*. The E3 ligases and substrate proteins were represented by *x* and *y*, respectively, and protein ubiquitination was denoted by (*x*, *y*) ∈ *E* to indicate the recognition of a specific substrate *y* by E3 ligase *x* (Additional file 2: Figure S1). Due to limited knowledge about ubiquitinated substrates that are recognized by E3 ligases, (*x*, *y*) could also represent a type of protein-protein interaction between E3 ligase *x* and ubiquitinated protein *y*. We used *V* to refer to all human proteins and *E*, to all experimentally confirmed PPIs. Cytoscape [58], a publicly available network viewer, was employed for the visualization of regulatory networks among E3 ligases and ubiquitinated substrates.

## Results and discussion

### Data statistics in this investigation

Data used for building the protein ubiquitination networks in this study were experimentally validated and supported with 39,814 research articles. Over 500 research articles were manually reviewed via a text mining method. In total, 41,392 ubiquitination sites from 12,786 ubiquitinated proteins in humans were extracted from 406 literatures. After removing redundant data among heterogeneous online resources, 494 experimentally verified human E3 ubiquitin ligases remained in the resulting data. PPIs between E3 ligases and ubiquitinated proteins were retrieved to deduce potential regulatory relationships between E3 ligases and ubiquitinated substrates to compensate for the limited information about E3 ligase targets. As shown in Table 1, 9,271 physical PPIs between 426 E3 ligases and 2,649 ubiquitinated proteins were curated. In particular, by incorporating the substrate motifs identified by the MDDLogo ubiquitination site prediction method [36]*,* potential substrates of E3 ligases could be inferred from the 27,227 PPIs between E3 ligases and other proteins. Moreover, 377,117 PPIs that appeared to involve ubiquitinated proteins could be integrated for the investigation of their functional associations in the context of ubiquitination.

**Table 1:** Data statistics in this work

Data content	Number of records
Ubiquitinated protein (potential E3 substrates)	12,786
Ubiquitination sites	41,392
E3 ubiquitin ligases	494
PPIs between E3 ligases and other proteins	27,227
PPIs between E3 ligases and ubiquitinated proteins	9,271
E3 ligases interacting with ubiquitinated proteins	436
Ubiquitinated proteins interacting with E3 ligases	2,649
Supported articles	39,814

### Substrate specificities of human ubiquitination sites

**Fig. 2:**
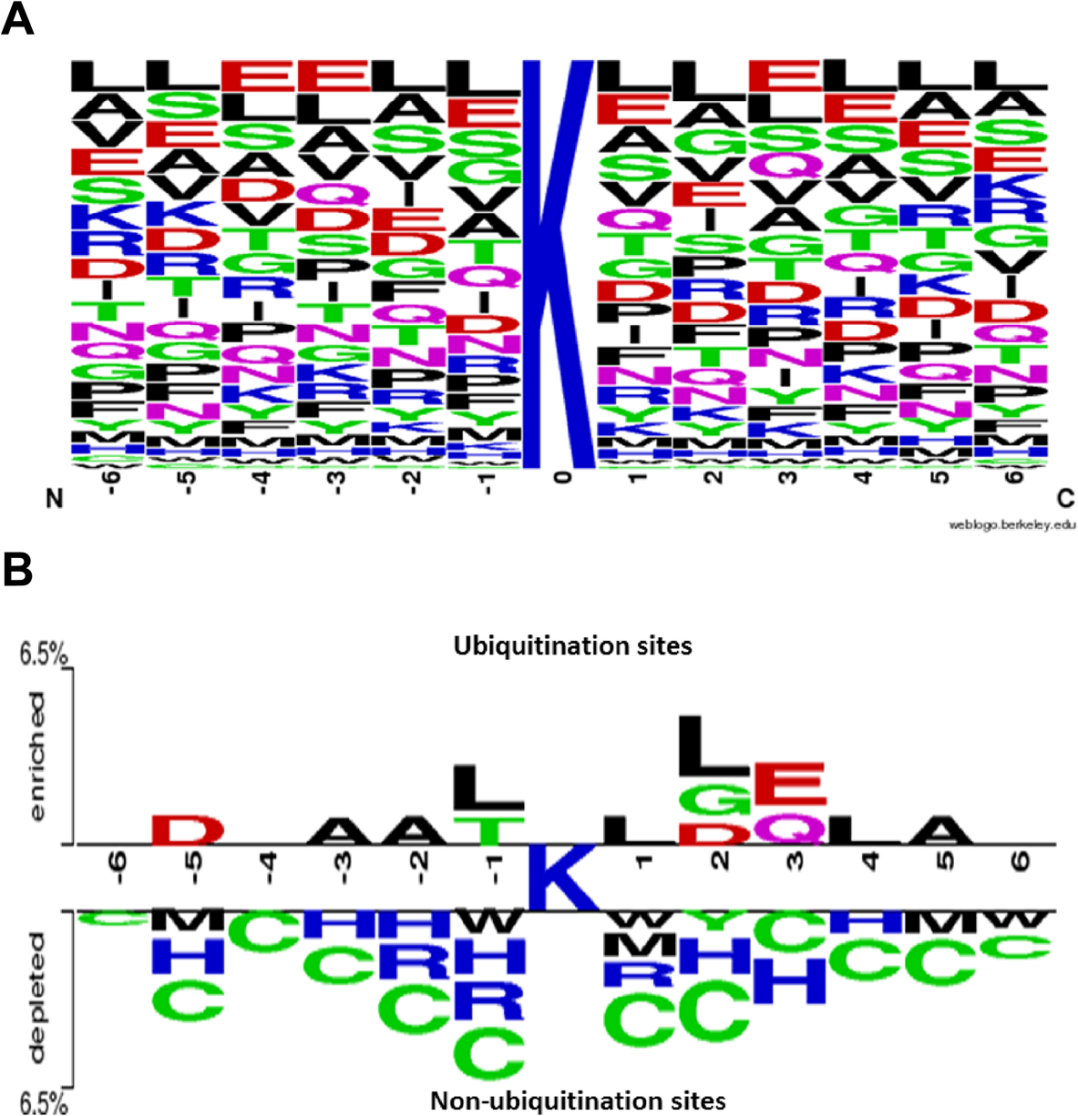
Amino acid composition of protein ubiquitination sites. **a** The frequency plot of ubiquitinated sites. **b** The compositional biases of amino acids around ubiquitination sites compared to the non-ubiquitination sites

The entropy plots generated by the sequence logo was used to graphically visualize the amino acid sequences flanking the substrate sites (at position 0). This allows for the easy observation of amino acid conservation surrounding the ubiquitination sites. Figure 2a shows Leu (L), Glu (E), and Ala (A) to be the most conserved amino acid residues as indicated by the position-specific amino aicd composition around the ubiquitinated lysines. Furthermore, using TwoSampleLogo, the differences in position-specific amino acid composition between ubiquitinated and non-ubiquitinated sites were revealed (Fig. 2b). The residues surrounding the ubiquitination sites were significantly enriched with Ala (A), Asp (D), Glu (E), Leu (L), Gly (G) and Thr (T), and depleted in Cys (C), His (H), Arg (R), Trp (W) and Met (M) (*p* < 0.005).

To overcome the difficulty of discovering conserved motifs from large-scale ubiquitinome data, the MDDLogo clustering method was adopted to search for substrate motifs from the curated human ubiquitination sites using a 13-mer window length. MDDLogo identified a total of nine subgroups containing conserved motifs from non-homologous human ubiquitination sites (Additional file 3: Table S2). While subgroup 1 (241 ubiquitination sites) contained the conserved amino acid composition at positions +3 and +5, the conserved motif of subgroup 2 included Arginine (R), Lysine (K), Phenylalanine (F), Tyrosine (Y) and Tryptophan (W) residues at position +5. The conserved motifs of Subgroups 3 and 8 comprised Glutamic acid (E), Aspartic acid (D), Glutamine (Q) and Asparagine (N) residues at positions +3 and -2, respectively. In contrast, the remaining subgroups consisted of Phenylalanine (F), Tyrosine (Y) and Tryptophan (W) residues at various specific positions in their conserved motifs. Thus, substrate motifs for ubiquitination sites may be determined by the position-specific conservation of Phenylalanine (F), Tyrosine (Y) and Tryptophan (W) residues. Furthermore, MDDLogo could be utilized to identify putative ubiquitination sites and potential interaction between E3 ligase and ubiquitinated proteins based on substrate motif conservation.

### Functional associations of E3 ligases and ubiquitinated proteins

Distributions of GO annotations for E3 ligases and ubiquitinated proteins categorized according to their corresponding biological processes, molecular functions and cellular components are provided in (Additional file 4: Table S3 and Additional file 5: Table S4), respectively. Following the InterPro annotation, the most abundant protein domain for E3 ligases appeared to belong to the “Zinc finger, RING-type RNA” (Table 2). In a genome-wide study of E3 ligases, it was suggested that the mammalian genomes encode more than 600 potential RING finger E3s [59]. E3 ligases containing the RING finger domain facilitate the interaction between an E2 enzyme and a substrate to mediate the transfer of ubiquitin from E2 to the target [60, 61]. On the other hand, those with the HECT domain are involved in the regulation of cellular trafficking, immune response, cellular growth and proliferation [62]. The HECT domain containing E3 ligases form a catalytic intermediate with ubiquitin and is responsible for the catalysis of two reactions: 1) transesterification reaction, in which ubiquitin from the cysteine residue at the E2 active site is transferred to another cysteine residue in the HECT domain [60]; 2) the subsequent attack of a substrate lysine on the thioester of the ubiquitin-bound HECT domain [63] (Additional file 6: Figure S2). Whereas the C-terminus of the HECT domain is more conserved, the N-terminus, the part that mediates substrate targeting, is more diverse [62].

**Table 2:** The distribution of top 20 functional domains for human E3 ligases

No.	InterPro ID	Domain terms	Number of proteins	% Total
1	IPR001680	WD40 repeat	287	57.5150 %
2	IPR006652	Kelch repeat type 1	68	13.6273 %
3	IPR000408	Regulator of chromosome condensation, RCC1	55	11.0220 %
4	IPR001841	Zinc finger, RING-type	54	10.8216 %
5	IPR000315	Zinc finger, B-box	48	9.6192 %
6	IPR003877	SPla/RYanodine receptor SPRY	38	7.6152 %
7	IPR013069	BTB/POZ	28	5.6112 %
8	IPR000569	HECT	28	5.6112 %
9	IPR001202	WW/Rsp5/WWP	28	5.6112 %
10	IPR020683	Ankyrin repeat-containing domain	27	5.4108 %
11	IPR001496	SOCS protein, C-terminal	25	5.0100 %
12	IPR002867	Zinc finger, C6HC-type	23	4.6092 %
13	IPR018957	Zinc finger, C3HC4 RING-type	22	4.4088 %
14	IPR001258	NHL repeat	21	4.2084 %
15	IPR011705	BTB/Kelch-associated	15	3.0060 %
16	IPR002110	Ankyrin repeat	14	2.8056 %
17	IPR000571	Zinc finger, CCCH-type	11	2.2044 %
18	IPR001876	Zinc finger, RanBP2-type	11	2.2044 %
19	IPR011016	Zinc finger, RING-CH-type	11	2.2044 %
20	IPR001452	Src homology-3 domain	10	2.0040 %

According to the annotation information on InterPro, approximately 70 % of established ubiquitination sites are mapped to specific functional domains, suggesting that ubiquitination may modulate a variety of biological functions. The top 50 InterPro functional domains containing ubiquitinated sites in humans are given in Table 3. It appeared that most ubiquitination sites could be found in the MHC class I (alpha chain) protein domains. It has been reported that viral proteins could induce the degradation of the histocompatibility complex (MHC) class I protein in the endoplasmic reticulum and at the cell surface by ubiquitinating the MHC class I domain [64]. The immunoglobulin C1-set domain, or classical Ig-like domains that resemble the antibody constant domain, is another domain found to be enriched with ubiquitinated sites. Interestingly, these domains were found exclusively in mediators of immune response, including various T-cell receptors, MHC class I and II complexes [65].

**Table 3:** Distribution of the top 50 functional domains covering ubiquitination sites

No.	Domain (InterPro) ID	Domain (InterPro) terms	Number of sites	% Total
1	IPR001039	MHC class I, alpha chain, alpha1/alpha2	1625	3.6574 %
2	IPR003597	Immunoglobulin C1-set	1321	2.9732 %
3	IPR001680	WD40 repeat	1043	2.3475 %
4	IPR002017	Spectrin repeat	1012	2.2777 %
5	IPR010579	MHC class I, alpha chain, C-terminal	729	1.6408 %
6	IPR003961	Fibronectin, type III	469	1.0556 %
7	IPR000504	RNA recognition motif domain	419	0.9431 %
8	IPR000719	Protein kinase, catalytic domain	338	0.7607 %
9	IPR013098	Immunoglobulin I-set	232	0.5222 %
10	IPR020683	Ankyrin repeat-containing domain	230	0.5177 %
11	IPR017868	Filamin/ABP280 repeat-like	212	0.4772 %
12	IPR006209	EGF-like domain	179	0.4029 %
13	IPR001715	Calponin homology domain	177	0.3984 %
14	IPR010630	Neuroblastoma breakpoint family (NBPF) domain	168	0.3781 %
15	IPR001452	Src homology-3 domain	167	0.3759 %
16	IPR006652	Kelch repeat type 1	158	0.3556 %
17	IPR000408	Regulator of chromosome condensation, RCC1	157	0.3534 %
18	IPR000225	Armadillo	156	0.3511 %
19	IPR004088	K Homology domain, type 1	154	0.3466 %
20	IPR001650	Helicase, C-terminal	146	0.3286 %
21	IPR002126	Cadherin	145	0.3264 %
22	IPR000048	IQ motif, EF-hand binding site	143	0.3219 %
23	IPR000083	Fibronectin, type I	143	0.3219 %
24	IPR000626	Ubiquitin	142	0.3196 %
25	IPR000008	C2 calcium-dependent membrane targeting	135	0.3038 %
26	IPR001806	Small GTPase superfamily	125	0.2813 %
27	IPR001245	Serine-threonine/tyrosine-protein kinase catalytic domain	125	0.2813 %
28	IPR000433	Zinc finger, ZZ-type	123	0.2768 %
29	IPR001478	PDZ domain	122	0.2746 %
30	IPR001849	Pleckstrin homology domain	120	0.2701 %
31	IPR011545	DNA/RNA helicase, DEAD/DEAH box type, N-terminal	112	0.2521 %
32	IPR019787	Zinc finger, PHD-finger	111	0.2498 %
33	IPR001440	Tetratricopeptide TPR-1	111	0.2498 %
34	IPR001202	WW/Rsp5/WWP	110	0.2476 %
35	IPR018108	Mitochondrial substrate/solute carrier	110	0.2476 %
36	IPR001781	Zinc finger, LIM-type	106	0.2386 %
37	IPR001101	Plectin repeat	104	0.2341 %
38	IPR002049	EGF-like, laminin	100	0.2251 %
39	IPR002429	Cytochrome c oxidase subunit II C-terminal	96	0.2161 %
40	IPR011759	Cytochrome C oxidase subunit II, transmembrane domain	96	0.2161 %
41	IPR003439	ABC transporter-like	91	0.2048 %
42	IPR001487	Bromodomain	91	0.2048 %
43	IPR018502	Annexin repeat	88	0.1981 %
44	IPR000980	SH2 domain	86	0.1936 %
45	IPR003008	Tubulin/FtsZ, GTPase domain	77	0.1733 %
46	IPR001421	ATPase, F0 complex, subunit 8, mitochondrial, Metazoan	75	0.1688 %
47	IPR013069	BTB/POZ	75	0.1688 %
48	IPR003959	ATPase, AAA-type, core	74	0.1666 %
49	IPR007087	Zinc finger, C2H2	74	0.1666 %
50	IPR000197	Zinc finger, TAZ-type	74	0.1666 %

### Network analysis for a group of interested proteins

To allow users to efficiently search for the proteins of their interest, a convenient interactive network viewer was implemented in the proposed method implemented an interactive network viewer. An example of constructing a protein ubiquitination network using our approach is illustrated in (Additional file 7: Figure S3). The network was built with four E3 ligases, 14 ubiquitinated proteins and three other proteins. While the established interactions between the four E3 ligases and 14 ubiquitinated proteins were immediately recognized, three other proteins interacting with two of the E3 ligases were also revealed as potential ubiquitinated substrates. For instance, E3 ligase MDM2 was predicted to target Forkhead box protein O3 (FOXO3) for ubiquitination. This is consistent with a recent study supporting MDM2 to be an E3 ligase responsible for the ubiquitin-mediated degradation of FOXO3 [66]. As well, our approach could provide the potential ubiquitination sites and the corresponding substrate motifs for a specific protein. Furthermore, for a specific E3 ligase and their interacting ubiquitinated proteins, the analysis could even be extended to exploring their functional associations and creating a comprehensive ubiquitin regulatory network.

### A case study of the discovered E3 ligases associated with the regulation of p53

**Fig. 3:**
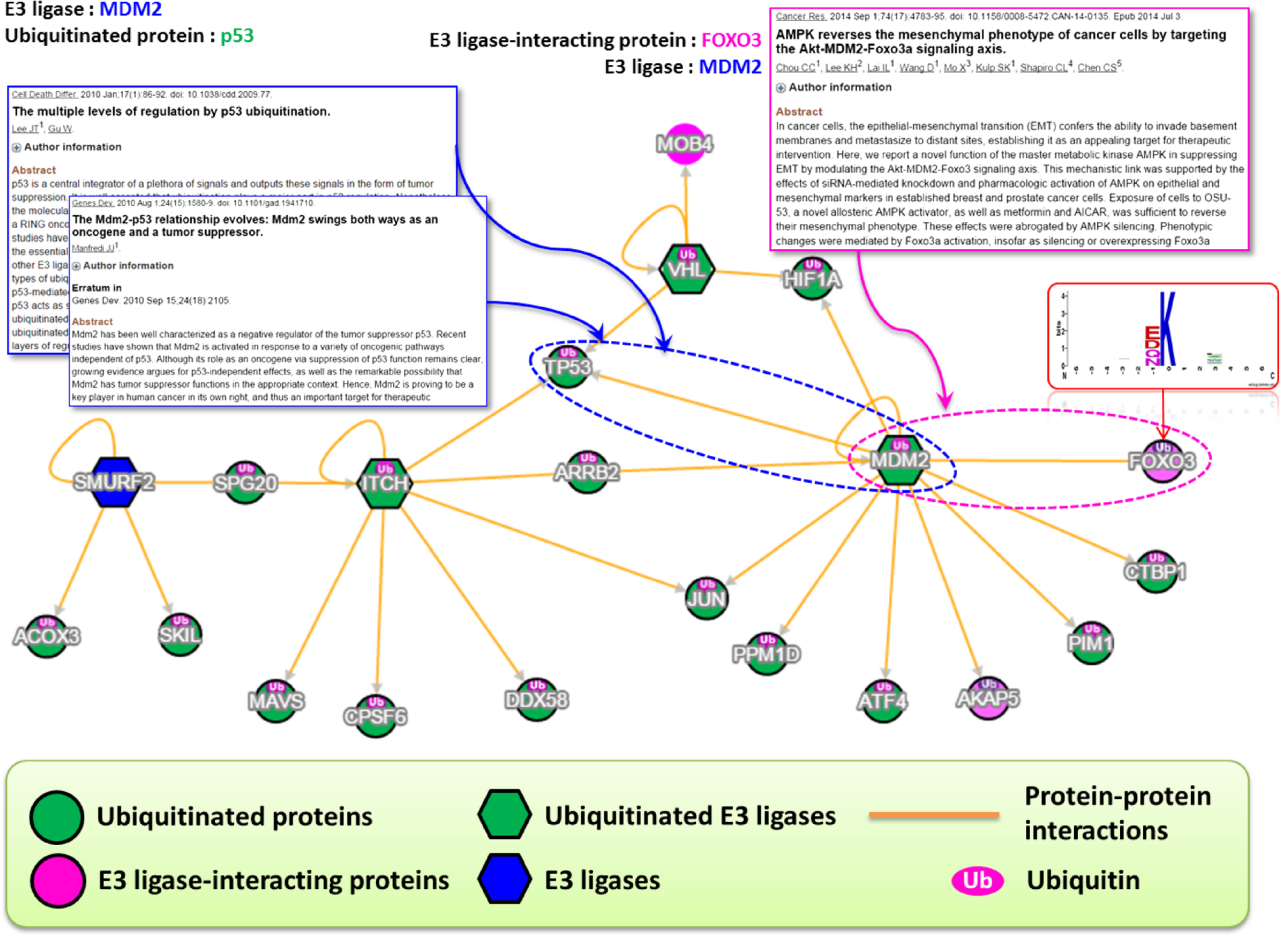
A case study of the discovered E3 ligases associated with the regulation of *tumor suppressor p53* (TP53)

In cases where information is limited with respect to the interaction between an E3 ligase and its corresponding substrates, our strategy could still identify the potential E3 ligases that may target a specific ubiquitinated protein. A case study is shown in Fig. 3, demonstrating the ability of the proposed method to construct an interaction map for the ubiquitination of tumor suppressor p53 (TP53). The resulting network is consistent with the literature. As a transcription factor, the tumor suppressor protein p53 responds to stress such as DNA damage by inducing cell cycle arrest and apoptosis [67]. Recent evidence has established that MDM2, a RING oncoprotein and a negative regulator of p53 [24], modulates the proteasomal degradation of p53 via a RING-finger-dependent manner [68–72]. Yet, our approach discovered other E3 ligases that may also regulate the ubiquitination of p53. Thus, the proposed strategy has the ability to uncover potential substrates for a specific E3 ligase, as well as potential E3 ligases for ubiquitinated proteins.

## Conclusion

In an attempt to characterize the regulatory role protein ubiquitination plays in a variety of biological processes, we combined the information of E3 ligases, ubiquitinated proteins, and protein-protein interactions to construct a comprehensive network of E3 ligases and their ubiquitinated substrates. Designed to serve as not only a meaningful platform for investigating E3-substrate regulatory networks but also a new strategy to uncover potential E3 ligases for ubiquitinated substrates, the proposed approach allows for the efficient characterization of protein ubiquitination networks from large-scale ubiquitinome data. With access to more updated data, the proposed scheme can be further refined for the study of E1 activating enzymes, E2 conjugating enzymes, and E3 ubiquitin ligases. Also, recent publications regarding the structural environment of experimentally validated ubiquitination sites based on protein tertiary structures [73–76] could be incorporated to infer the functional interactions between the enzymes and substrates. Finally, confirmed functional annotations of ubiquitination sites could be extracted from the literature via a more advanced information retrieval system to collect more adequate information required for further functional analyses.

## Additional files

Additional file 1: Table S1. Data statistics of protein-protein interactions obtained from public resources. (PDF 8 kb) Additional file 2: Figure S1. A conceptual diagram of exploiting Cytoscape software to construct protein ubiquitination networks based on graph theory. (PDF 86 kb) Additional file 3: Table S2. The entropy plot of sequence logos for MDDLogo-identified motifs obtained from non-homologous ubiquitination sites in humans. (PDF 86 kb) Additional file 4: Table S3. Distribution of the top 15 GO annotations for human E3 ligases. (PDF 31 kb) Additional file 5: Table S4. Distribution of the top 20 GO annotations for human ubiquitinated proteins. (PDF 52 kb) Additional file 6: Figure S2. The HECT-type structure of E3 ligases. (PDF 133 kb) Additional file 7: Figure S3. An example to construct protein ubiquitination network for 21 proteins (containing four E3 ligases and 14 ubiquitinated proteins). (PDF 345 kb)
