# Supplementary material for: A new scheme to discover functional associations and regulatory networks of E3 ubiquitin ligases
Source: BMC Syst Biol. 2016 Jan 11;10(Suppl 1):3. doi: 10.1186/s12918-015-0244-1 (PMC4895279; doi:10.1186/s12918-015-0244-1)
Supplement: Additional file 1: Table S1. — Data statistics of protein-protein interactions obtained from public resources. (PDF 8 kb) [file 12918_2015_244_MOESM1_ESM.pdf]

Table S1. Data statistics of protein-protein interactions obtained from public resources.

PPI Resource	# Data records
Reactome	245659
iRefIndex	103479
APID	78210
IntAct	45971
MINT	21473
Spike	20164
Interoporc	18638
DIP	13099
UniProt	2962
I2DIMex	398
MolCon	177
MatrixDB	171
MBInfo	123
**Combined data (Unique PPIs)**	**418891**
