# Supplementary material for: A new scheme to discover functional associations and regulatory networks of E3 ubiquitin ligases
Source: BMC Syst Biol. 2016 Jan 11;10(Suppl 1):3. doi: 10.1186/s12918-015-0244-1 (PMC4895279; doi:10.1186/s12918-015-0244-1)
Supplement: Additional file 2: Figure S1. — A conceptual diagram of exploiting Cytoscape software to construct protein ubiquitination networks based on graph theory. (PDF 86 kb) [file 12918_2015_244_MOESM2_ESM.pdf]

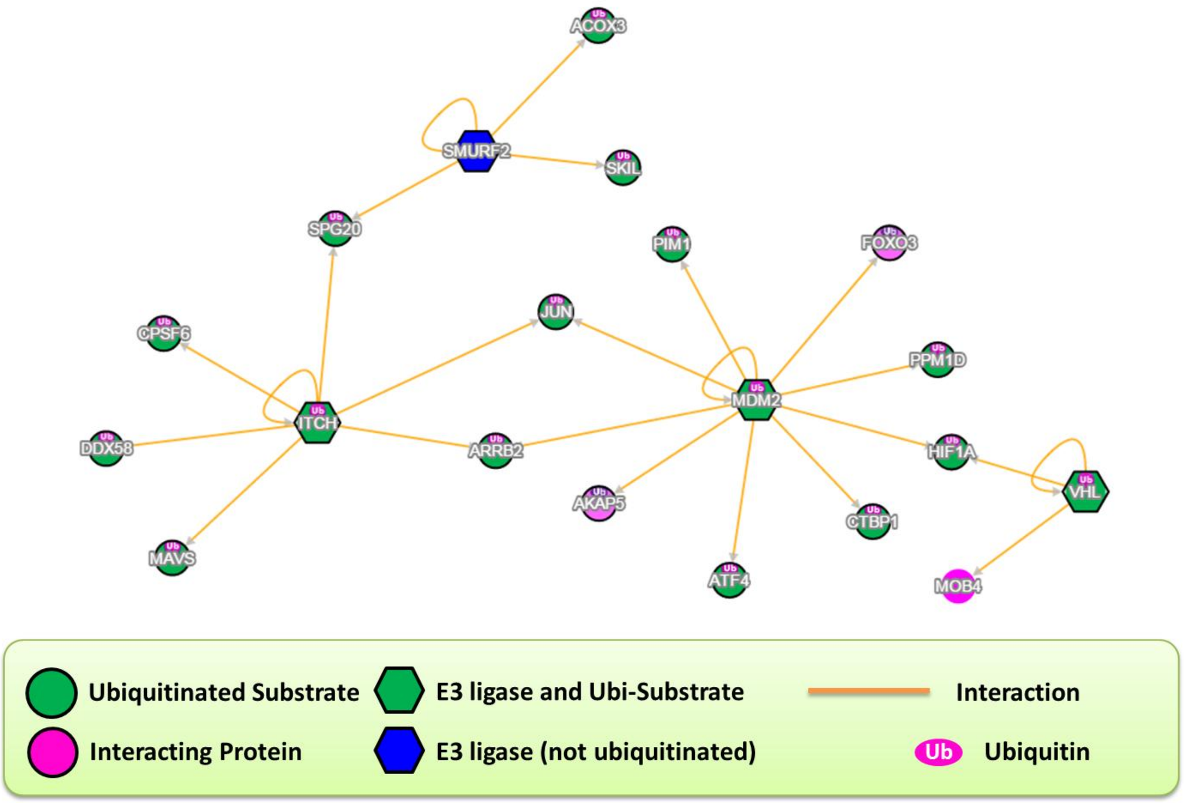

Figure S1. A conceptual diagram of exploiting Cytoscape software to construct protein ubiquitination networks based on graph theory.
