# Supplementary material for: A new scheme to discover functional associations and regulatory networks of E3 ubiquitin ligases
Source: BMC Syst Biol. 2016 Jan 11;10(Suppl 1):3. doi: 10.1186/s12918-015-0244-1 (PMC4895279; doi:10.1186/s12918-015-0244-1)
Supplement: Additional file 3: Table S2. — The entropy plot of sequence logos for MDDLogo-identified motifs obtained from non-homologous ubiquitination sites in humans. (PDF 86 kb) [file 12918_2015_244_MOESM3_ESM.pdf]

Table S2. The entropy plot of sequence logos for MDDLogo-identified motifs obtained from non-homologous ubiquitination sites in humans.

Group	Number of ubiquitination sites	Entropy plot of MDDLogo-identified motifs
1	241	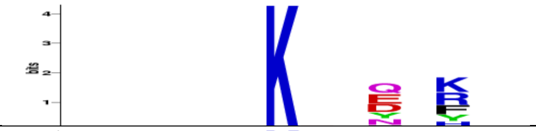
2	452	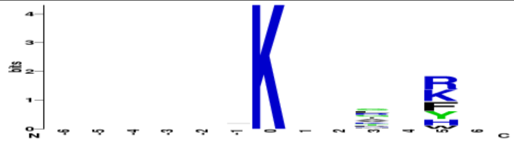
3	506	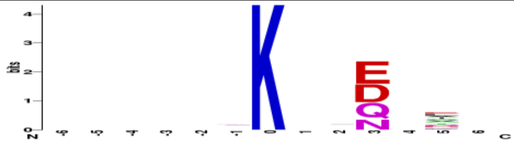
4	145	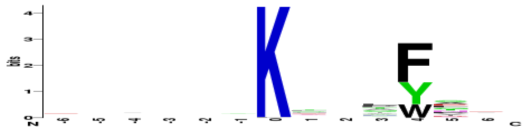
5	160	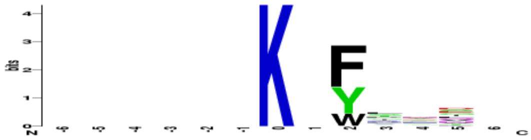
6	136	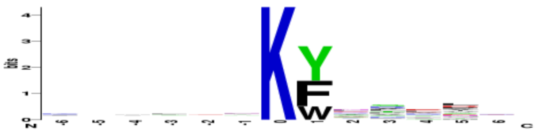
7	108	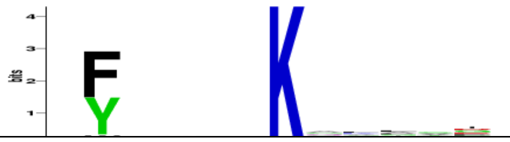
8	237	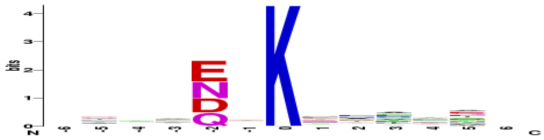
9	73	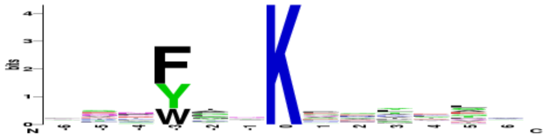
10	583	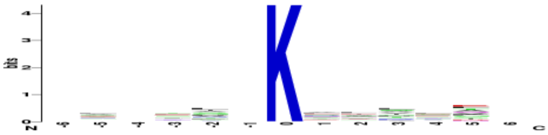
