# Supplementary material for: A new scheme to discover functional associations and regulatory networks of E3 ubiquitin ligases
Source: BMC Syst Biol. 2016 Jan 11;10(Suppl 1):3. doi: 10.1186/s12918-015-0244-1 (PMC4895279; doi:10.1186/s12918-015-0244-1)
Supplement: Additional file 4: Table S3. — Distribution of the top 15 GO annotations for human E3 ligases. (PDF 31 kb) [file 12918_2015_244_MOESM4_ESM.pdf]

**Table S3. Distribution of the top 15 GO annotations for human E3 ligases.**

| No. | Gene Ontology (GO) ID | Gene Ontology (GO) Terms | Number of E3s | % Total |
|---|---|---|---|---|
| **Biological Processes** | | | | |
| 1 | GO:0048523 | negative regulation of cellular process | 9 | 1.80% |
| 2 | GO:0048518 | positive regulation of biological process | 8 | 1.60% |
| 3 | GO:0048522 | positive regulation of cellular process | 8 | 1.60% |
| 4 | GO:0048519 | negative regulation of biological process | 8 | 1.60% |
| 5 | GO:0019222 | regulation of metabolic process | 8 | 1.60% |
| 6 | GO:0050794 | regulation of cellular process | 7 | 1.40% |
| 7 | GO:0010604 | positive regulation of macromolecule metabolic process | 7 | 1.40% |
| 8 | GO:0050789 | regulation of biological process | 7 | 1.40% |
| 9 | GO:0031325 | positive regulation of cellular metabolic process | 7 | 1.40% |
| 10 | GO:0031323 | regulation of cellular metabolic process | 7 | 1.40% |
| 11 | GO:0065007 | biological regulation | 7 | 1.40% |
| 12 | GO:0060255 | regulation of macromolecule metabolic process | 7 | 1.40% |
| 13 | GO:0080090 | regulation of primary metabolic process | 7 | 1.40% |
| 14 | GO:0007049 | cell cycle | 6 | 1.20% |
| 15 | GO:0023033 | signaling pathway | 6 | 1.20% |
| **Cellular Localization** | | | | |
| 1 | GO:0005634 | nucleus | 12 | 2.40% |
| 2 | GO:0005829 | cytosol | 11 | 2.20% |
| 3 | GO:0005768 | endosome | 7 | 1.40% |
| 4 | GO:0043229 | intracellular organelle | 7 | 1.40% |
| 5 | GO:0043226 | organelle | 7 | 1.40% |
| 6 | GO:0005654 | nucleoplasm | 6 | 1.20% |
| 7 | GO:0044428 | nuclear part | 6 | 1.20% |
| 8 | GO:0044446 | intracellular organelle part | 6 | 1.20% |
| 9 | GO:0044422 | organelle part | 6 | 1.20% |
| 10 | GO:0043231 | intracellular membrane-bounded organelle | 6 | 1.20% |
| 11 | GO:0043227 | membrane-bounded organelle | 6 | 1.20% |
| 12 | GO:0005737 | cytoplasm | 6 | 1.20% |
| 13 | GO:0044424 | intracellular part | 6 | 1.20% |
| 14 | GO:0010008 | endosome membrane | 5 | 1.00% |
| 15 | GO:0044440 | endosomal part | 5 | 1.00% |
| **Molecular Function** | | | | |
| 1 | GO:0005515 | protein binding | 11 | 2.20% |
| 2 | GO:0016563 | transcription activator activity | 7 | 1.40% |
| 3 | GO:0019899 | enzyme binding | 6 | 1.20% |
| 4 | GO:0008134 | transcription factor binding | 6 | 1.20% |
| 5 | GO:0030528 | transcription regulator activity | 6 | 1.20% |
| 6 | GO:0003713 | transcription coactivator activity | 5 | 1.00% |
| 7 | GO:0004716 | receptor signaling protein tyrosine kinase activity | 5 | 1.00% |

| No. | Gene Ontology (GO) ID | Gene Ontology (GO) Terms | Number of E3s | % Total |
|---|---|---|---|---|
| 8 | GO:0005057 | receptor signaling protein activity | 5 | 1.00% |
| 9 | GO:0004871 | signal transducer activity | 4 | 0.80% |
| 10 | GO:0060089 | molecular transducer activity | 4 | 0.80% |
| 11 | GO:0004888 | transmembrane receptor activity | 4 | 0.80% |
| 12 | GO:0004872 | receptor activity | 4 | 0.80% |
| 13 | GO:0010843 | promoter binding | 4 | 0.80% |
| 14 | GO:0044212 | DNA regulatory region binding | 4 | 0.80% |
| 15 | GO:0004713 | protein tyrosine kinase activity | 3 | 0.60% |
