# Supplementary material for: A new scheme to discover functional associations and regulatory networks of E3 ubiquitin ligases
Source: BMC Syst Biol. 2016 Jan 11;10(Suppl 1):3. doi: 10.1186/s12918-015-0244-1 (PMC4895279; doi:10.1186/s12918-015-0244-1)
Supplement: Additional file 5: Table S4. — Distribution of the top 20 GO annotations for human ubiquitinated proteins. (PDF 52 kb) [file 12918_2015_244_MOESM5_ESM.pdf]

Table S4. Distribution of the top 20 GO annotations for human ubiquitinated proteins.

#	Gene Ontology (GO) ID	Gene Ontology (GO) Terms	Number of proteins	% Total	*p*-Value
**Biological Processes**					
1	GO:0046907	intracellular transport	423	6.89	1.85E-50
2	GO:0007049	cell cycle	476	7.76	4.64E-48
3	GO:0000278	mitotic cell cycle	268	4.37	4.98E-46
4	GO:0044265	cellular macromolecule catabolic process	447	7.29	6.69E-46
5	GO:0045184	establishment of protein localization	465	7.58	1.64E-44
6	GO:0022402	cell cycle process	366	5.97	1.86E-44
7	GO:0015031	protein transport	461	7.51	3.35E-44
8	GO:0008104	protein localization	516	8.41	1.18E-43
9	GO:0009057	macromolecule catabolic process	467	7.61	7.22E-43
10	GO:0006412	translation	241	3.93	5.06E-42
11	GO:0051603	proteolysis involved in cellular protein catabolic process	376	6.13	8.63E-41
12	GO:0043632	modification-dependent macromolecule catabolic process	363	5.92	1.24E-40
13	GO:0019941	modification-dependent protein catabolic process	363	5.92	1.24E-40
14	GO:0030163	protein catabolic process	386	6.29	1.81E-40
15	GO:0044257	cellular protein catabolic process	376	6.13	4.41E-40
16	GO:0065003	macromolecular complex assembly	397	6.47	3.96E-36
17	GO:0006414	translational elongation	96	1.56	7.13E-35
18	GO:0043933	macromolecular complex subunit organization	415	6.76	7.84E-35
19	GO:0034613	cellular protein localization	269	4.38	8.52E-34
20	GO:0070727	cellular macromolecule localization	270	4.40	1.58E-33
**Molecular Function**					
1	GO:0000166	nucleotide binding	1193	19.45	3.52E-78
2	GO:0032555	purine ribonucleotide binding	976	15.91	5.61E-62
3	GO:0032553	ribonucleotide binding	976	15.91	5.61E-62
4	GO:0017076	purine nucleotide binding	1007	16.41	1.39E-60
5	GO:0032559	adenyl ribonucleotide binding	801	13.06	3.23E-51
6	GO:0001882	nucleoside binding	850	13.85	4.32E-51
7	GO:0005524	ATP binding	791	12.89	1.01E-50
8	GO:0001883	purine nucleoside binding	843	13.74	2.75E-50
9	GO:0030554	adenyl nucleotide binding	832	13.56	5.02E-50
10	GO:0003723	RNA binding	438	7.14	9.43E-46
11	GO:0016887	ATPase activity	209	3.41	6.72E-24
12	GO:0016881	acid-amino acid ligase activity	142	2.31	8.85E-24
13	GO:0003735	structural constituent of ribosome	124	2.02	1.54E-23
14	GO:0016879	ligase activity, forming carbon-nitrogen bonds	157	2.56	1.95E-23
15	GO:0019787	small conjugating protein ligase activity	122	1.99	6.66E-23
16	GO:0042623	ATPase activity, coupled	174	2.84	1.66E-21

17	GO:0004842	ubiquitin-protein ligase activity	108	1.76	2.45E-20
18	GO:0008134	transcription factor binding	277	4.52	3.98E-18
19	GO:0019899	enzyme binding	279	4.55	2.47E-17
20	GO:0004386	helicase activity	96	1.56	6.50E-15
**Cellular Component**					
1	GO:0070013	intracellular organelle lumen	1060	17.28	2.92E-119
2	GO:0031974	membrane-enclosed lumen	1085	17.69	1.58E-114
3	GO:0005829	cytosol	843	13.74	4.30E-113
4	GO:0043233	organelle lumen	1065	17.36	1.81E-112
5	GO:0043232	intracellular non-membrane-bounded organelle	1383	22.54	1.10E-106
6	GO:0043228	non-membrane-bounded organelle	1383	22.54	1.10E-106
7	GO:0031981	nuclear lumen	882	14.38	7.42E-104
8	GO:0005654	nucleoplasm	562	9.16	3.68E-74
9	GO:0030529	ribonucleoprotein complex	355	5.79	1.88E-58
10	GO:0005694	chromosome	303	4.94	1.66E-43
11	GO:0031975	envelope	378	6.16	4.44E-42
12	GO:0031967	organelle envelope	377	6.15	4.78E-42
13	GO:0005730	nucleolus	412	6.72	1.54E-41
14	GO:0044427	chromosomal part	257	4.19	4.30E-38
15	GO:0005739	mitochondrion	565	9.21	1.01E-34
16	GO:0031090	organelle membrane	568	9.26	1.88E-34
17	GO:0044429	mitochondrial part	334	5.44	5.54E-28
18	GO:0005840	ribosome	152	2.48	6.27E-27
19	GO:0015630	microtubule cytoskeleton	310	5.05	1.41E-26
20	GO:0022626	cytosolic ribosome	74	1.21	1.75E-25
