# Supplementary figures and images for: A new scheme to discover functional associations and regulatory networks of E3 ubiquitin ligases
Source: BMC Syst Biol. 2016 Jan 11;10(Suppl 1):3. doi: 10.1186/s12918-015-0244-1 (PMC4895279; doi:10.1186/s12918-015-0244-1)

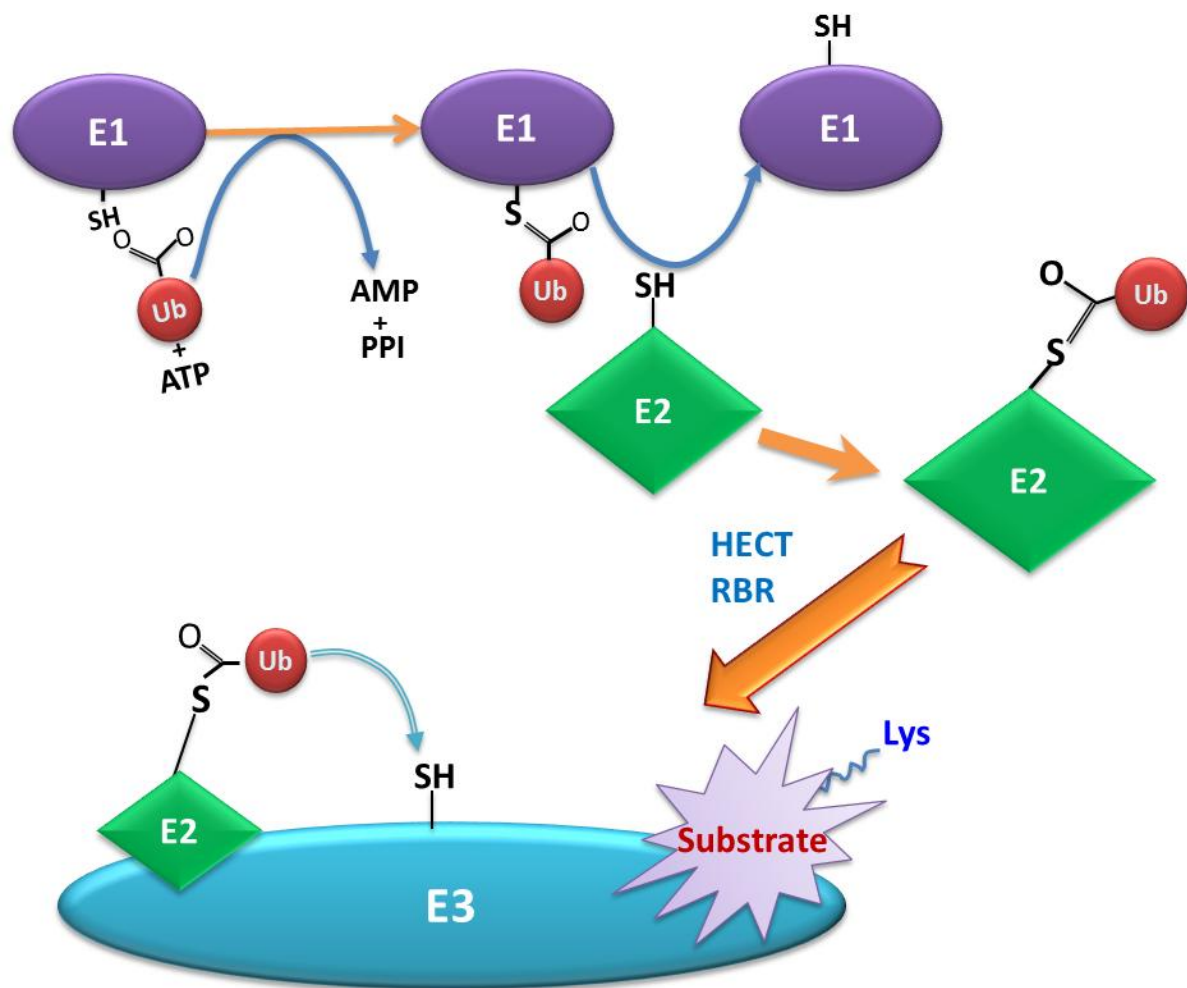

Figure S2. The HECT-type structure of E3 ligases.

Supplement: Additional file 6: Figure S2. — The HECT-type structure of E3 ligases. (PDF 133 kb) [file 12918_2015_244_MOESM6_ESM.pdf]
