# Supplementary material for: A new scheme to discover functional associations and regulatory networks of E3 ubiquitin ligases
Source: BMC Syst Biol. 2016 Jan 11;10(Suppl 1):3. doi: 10.1186/s12918-015-0244-1 (PMC4895279; doi:10.1186/s12918-015-0244-1)
Supplement: Additional file 7: Figure S3. — An example to construct protein ubiquitination network for 21 proteins (containing four E3 ligases and 14 ubiquitinated proteins). (PDF 345 kb) [file 12918_2015_244_MOESM7_ESM.pdf]

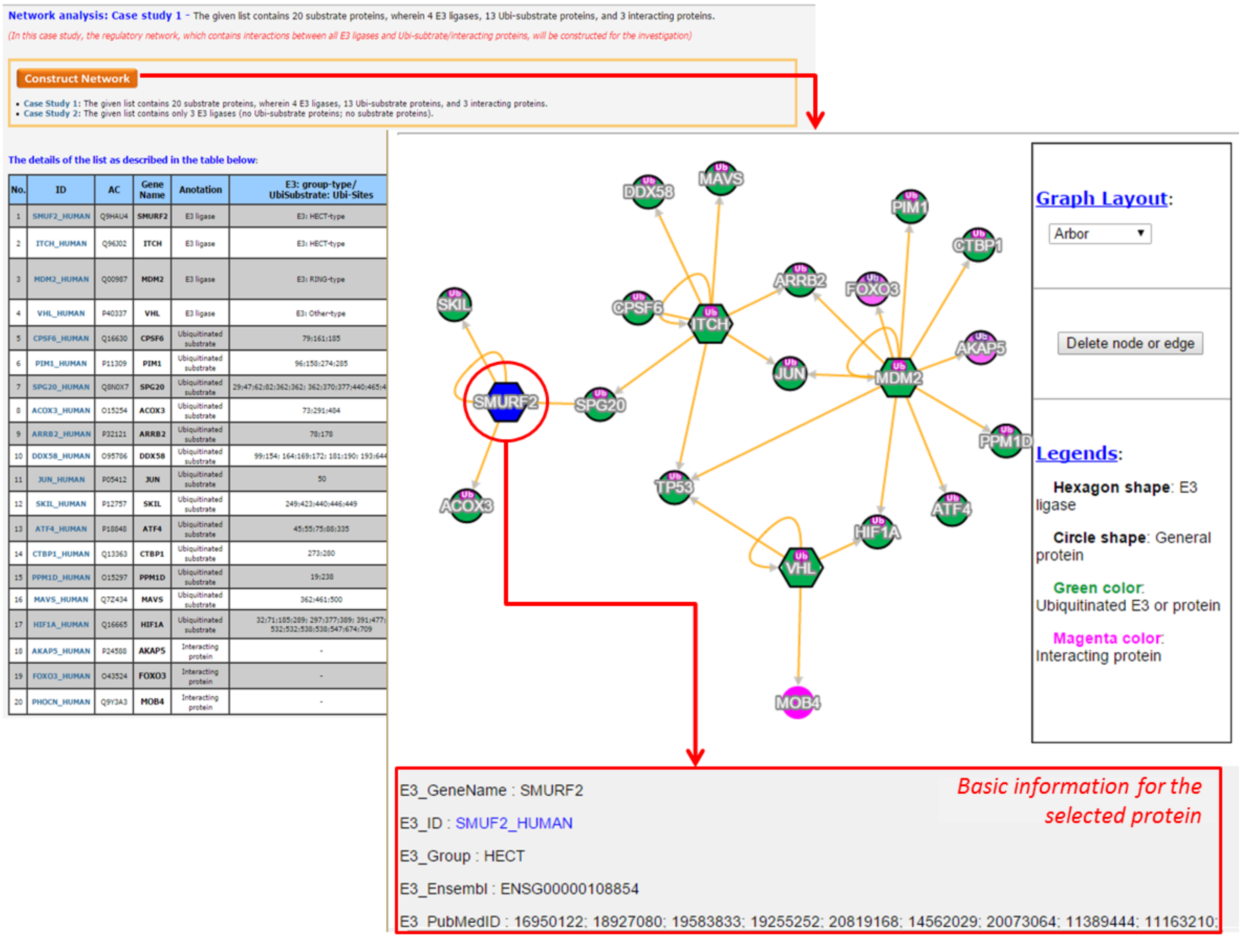

Figure S3. An example to construct protein ubiquitination network for 21 proteins (containing four E3 ligases and 14 ubiquitinated proteins).
